# A Novel System for the Device-Based Measurement of Physical Activity, Sedentary Behavior, and Sleep (Motus): Usability Evaluation

**DOI:** 10.2196/48209

**Published:** 2023-11-17

**Authors:** Patrick Crowley, Rasmus Kildedal, Simon Overvad Vindelev, Sandra Schade Jacobsen, Jon Roslyng Larsen, Peter J Johansson, Mette Aadahl, Leon Straker, Emmanuel Stamatakis, Andreas Holtermann, Paul Jarle Mork, Nidhi Gupta

**Affiliations:** 1 The National Research Centre for the Working Environment Copenhagen Denmark; 2 Department of Medical Sciences, Occupational and Environmental Medicine Uppsala University Uppsala Sweden; 3 Occupational and Environmental Medicine Uppsala University Hospital Uppsala Sweden; 4 Center for Clinical Research and Prevention Bispebjerg and Frederiksberg Hospital Copenhagen Denmark; 5 Department of Clinical Medicine Faculty of Health and Medical Sciences University of Copenhagen Copenhagen Denmark; 6 School of Allied Health Curtin University Perth Australia; 7 Charles Perkins Centre Mackenzie Wearables Research Hub University of Sydney Sydney Australia; 8 School of Health Sciences Faculty of Medicine and Health University of Sydney Sydney Australia; 9 Department of Public Health and Nursing Faculty of Medicine and Health Sciences Norwegian University of Science and Technology Trondheim Norway

**Keywords:** usability, accelerometry, development, accelerometer, design, mhealth, mobile health, app, application, diary, thematic analysis, monitor, monitoring, physical activity, exercise, sedentary, sleep

## Abstract

**Background:**

Device-based measurements of physical behavior, using the current methods, place a large burden on participants. The Motus system could reduce this burden by removing the necessity for in-person meetings, replacing diaries written on paper with digital diaries, and increasing the automation of feedback generation.

**Objective:**

This study aims to describe the development of the Motus system and evaluate its potential to reduce participant burden in a two-phase usability evaluation.

**Methods:**

Motus was developed around (1) a thigh-worn accelerometer with Bluetooth data transfer; (2) a smartphone app containing an attachment guide, a digital diary, and facilitating automated data transfer; (3) a cloud infrastructure for data storage; (4) an analysis software to generate feedback for participants; and (5) a web-based app for administrators. We recruited 19 adults with a mean age of 45 (SD 11; range 27-63) years, of which 11 were female, to assist in the two-phase evaluation of Motus. A total of 7 participants evaluated the usability of mockups for a smartphone app in phase 1. Participants interacted with the app while thinking aloud, and any issues raised were classified as critical, serious, or minor by observers. This information was used to create an improved and functional smartphone app for evaluation in phase 2. A total of 12 participants completed a 7-day free-living measurement with Motus in phase 2. On day 1, participants attempted 20 system-related tasks under observation, including registration on the study web page, reading the information letter, downloading and navigating the smartphone app, attaching an accelerometer on the thigh, and completing a diary entry for both work and sleep hours. Task completion success and any issues encountered were noted by the observer. On completion of the 7-day measurement, participants provided a rating from 0 to 100 on the System Usability Scale and participated in a semistructured interview aimed at understanding their experience in more detail.

**Results:**

The task completion rate for the 20 tasks was 100% for 13 tasks, >80% for 4 tasks, and <50% for 3 tasks. The average rating of system usability was 86 on a 0-100 scale. Thematic analysis indicated that participants perceived the system as easy to use and remember, and subjectively pleasing overall. Participants with shift work reported difficulty with entering sleep hours, and 66% (8/12) of the participants experienced slow data transfer between the app and the cloud infrastructure. Finally, a few participants desired a greater degree of detail in the generated feedback.

**Conclusions:**

Our two-phase usability evaluation indicated that the overall usability of the Motus system is high in free-living. Issues around the system’s slow data transfer, participants with atypical work shifts, and the degree of automation and detail of generated feedback should be addressed in future iterations of the Motus system.

**International Registered Report Identifier (IRRID):**

RR2-10.2196/35697

## Introduction

The emergence of evidence highlighting the importance of multiple dimensions of physical behaviors [1,2] has placed new demands on the methods used to gather data [3,4]. The established shortcomings of self-report methods, such as recall bias and misclassification [5], will likely be exacerbated by these new demands [6]. For example, we can expect that the influence of recall bias will be greater for dimensions involving habitual behaviors (eg, postures such as standing and walking), which are even more difficult to recall than activities with discrete characteristics (eg, going for a run, cycling to work). We need to develop new and improved methods to provide an alternative, or supplement, to self-report.

Although device-based measurements (monitors worn on the body) have undergone considerable development over the past decades [7], their use in research still places a considerable burden on participants [3,8]. As highlighted by various previous studies [9-11], such burden on participants includes, for example, the requirement to meet in-person, restriction on certain activities during wear time, the requirement to complete and maintain paper diaries of work and sleep hours, and the delay in receiving meaningful feedback on their measurements.

The lack of suitable technologies has limited the development of less burdensome methods. More recently, the increased availability of more affordable sensors, the growth in ownership of internet-connected smartphones, and the accessibility of cloud computing have meant that we can now begin to develop methods with the potential to reduce the burden on participants [8]. The Motus system, developed as part of the SurPASS project [9], integrates many of the aforementioned newly accessible technologies to reduce the burden on participants. In theory, Motus should (1) remove the need for in-person meetings, (2) replace data collected on paper, and (3) automate the generation of participant feedback.

The aim of this work is to describe the development of the Motus system and evaluate the potential of Motus to reduce participant burden in a two-phase usability study.

## Methods

### Overview

**Figure 1:**
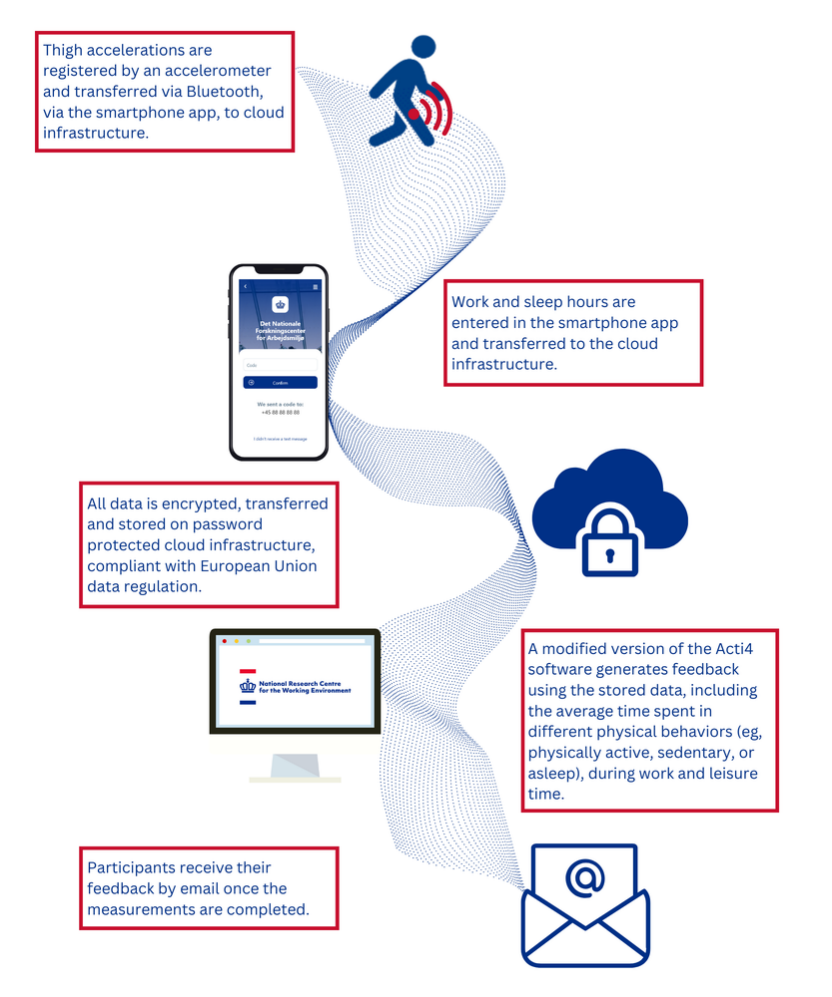
The data flow between the 5 core components of the Motus system. The data flows from the thigh-worn accelerometer, via the smartphone app, to cloud infrastructure.

The proposed protocol for the development and evaluation of the Motus system, with a detailed description of the system itself, has been published elsewhere [9]. In short, the Motus system consists of 5 core components, interacting as shown in Figure 1. Thigh accelerations are collected by the thigh-worn accelerometer, while data on participants’ work and sleep hours are entered daily in the smartphone app. All data are automatically transferred to the cloud infrastructure, where they can be accessed for analysis. Custom software then generates feedback (ie, summary statistics of the participant’s physical behaviors), which is then sent to the participant.

This evaluation focuses on the participant interaction with these core components, but also on the usability of the system as a whole from the participants’ perspective, which includes more procedural components such as registration on the study webpage and comprehension of recruitment material and the accelerometer attachment guide. Motus was developed by a project team of researchers at the National Research Center for the Working Environment working in close collaboration with industrial partners at SENS Innovation ApS. The development and evaluation were overseen and guided by both scientific and social reference groups [9].

### Ethical Considerations

Ethical approval for the SurPASS project was provided by the Scientific Ethics Committee of the Capital Region of Denmark (jr.n. 20030293) and data collection was conducted in accordance with the Declaration of Helsinki.

### Development and Evaluation Frameworks

System development was guided by the User-Centered Design framework [12] where design decisions are largely based on the participant’s needs and specifications [12]. System usability evaluation was guided by the System Acceptability Framework [13], which defines 5 subconstructs for the assessment of usability. Learnability is defined as the ease of use leading to the rapid attainment of proficiency. Efficiency is defined as the ease of use leading to high productivity once the system is learned. Memorability is defined as the ease of use leading to natural retention and removing the need to relearn. Issues is defined as the ease of use leading to few issues and easy recovery from any issues. Finally, satisfaction is defined as the ease of use leading to a pleasant and likable participant experience.

### Recruitment

**Figure 2:**
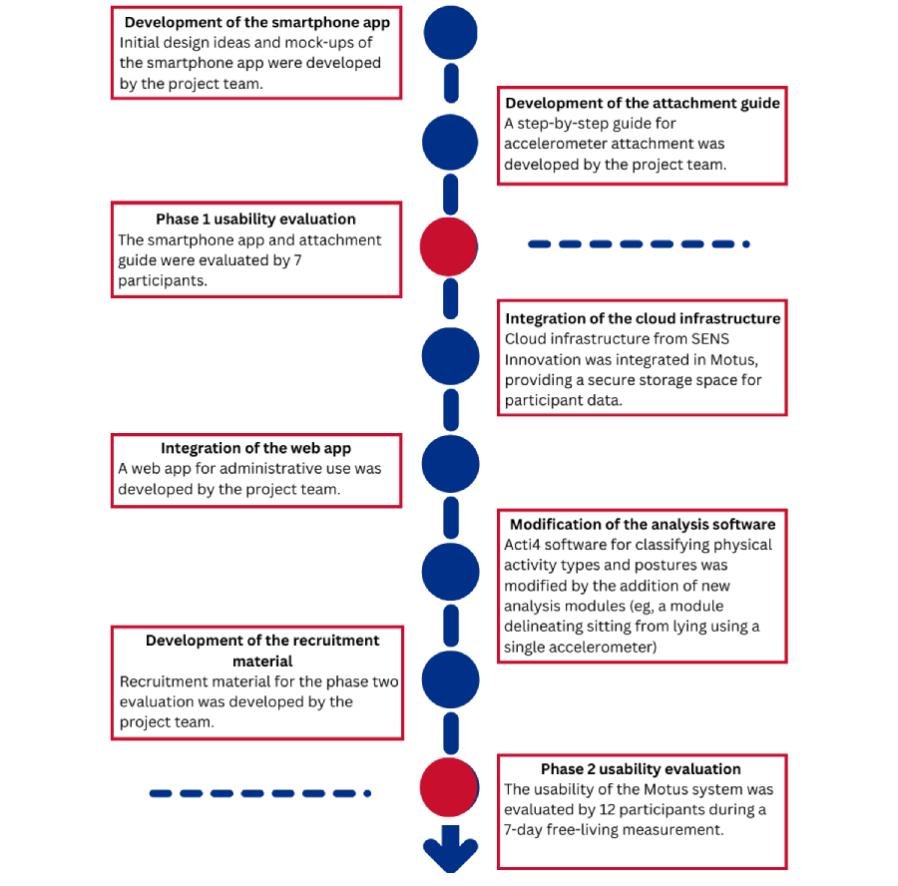
The development timeline of the Motus system, including the development of the smartphone app, the attachment guide, the back-end infrastructure, the web application for personnel use, the analysis software, and the recruitment material.

A total of 7 participants assisted in phase 1 evaluation (timeline shown in Figure 2). They were recruited as a convenience sample of nonacademic staff at the National Research Center for the Working Environment, Copenhagen. A total of 12 participants assisted in phase 2. They were recruited as a purposive sample through workplace union representatives, in contact with the National Research Center for the Working Environment. To increase the variety of participants we recruited based on variation in (1) job type (office, working with people, and industry workers), (2) shift type (workers with regular and irregular hours), (3) age, (4) education level, and (5) sex. Eligible participants had to own a smartphone and have access to a webcam, which was necessary for the digital meetings. Web-based meetings were conducted to ensure physical separation between the participant and the researcher, allowing participants to complete tasks in their own homes. The smartphone app used in phase 2 was developed initially for Android only. Samsung Galaxy S5 smartphones were loaned to participants who did not use Android phones.

### Phase 1 Development and Evaluation

#### Development of the Smartphone App

**Figure 3:**
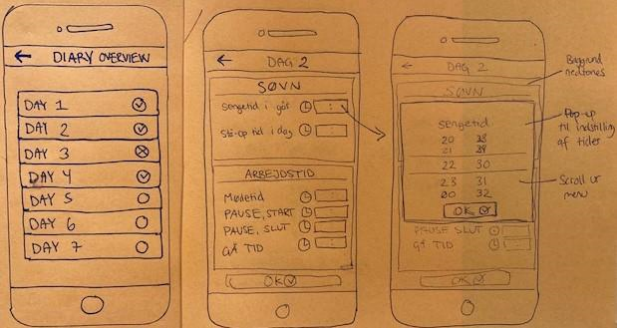
Examples of the initial design ideas illustrated on paper by the project team during the development of the app in phase 1. In this example, ideas for the layout of the diary app screens (from left to right) for the entry of work and sleep hours.

**Figure 4:**
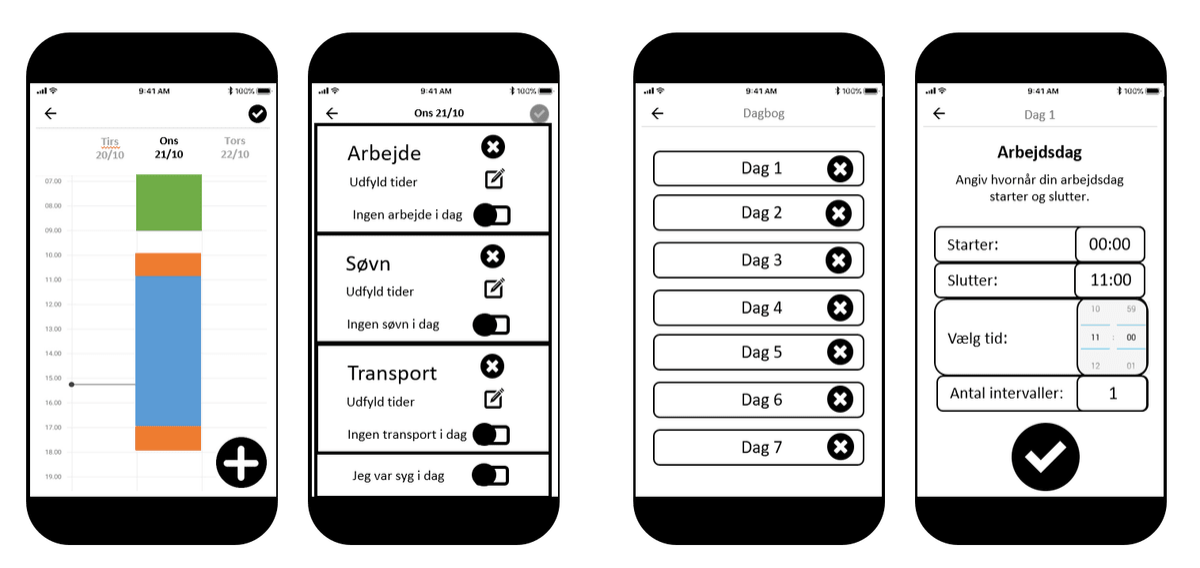
Examples of the initial digital mock-ups created in Microsoft PowerPoint by the project team during the development of the smartphone app in phase 1. From left to right, different ideas are presented for the layout of the diary app screens for the entry of work and sleep hours.

Initial design ideas for the layout of the smartphone were illustrated by the project team, first in paper mock-ups (see examples in Figure 3) and then in digital mock-ups using Microsoft PowerPoint (see examples in Figure 4).

The most promising mock-ups, judged by the project team, were converted to interactive browser-based app screens using Adobe XD (Adobe Inc), which were then presented to participants in evaluation phase 1 (described later). Using the information gathered during phase 1, a fully functional and improved smartphone app was developed using the Ionic 4 framework (Ionic). The Bluetooth data transfer component was written in Java (Oracle) for the Android version and Swift (Swift) for the IOS version of the app. App content was provided by researchers at the National Research Centre for the Working Environment, Copenhagen. Technical implementation and programming were conducted by SENS Innovation ApS. The fully functional app was used in the phase 2 evaluation (also described later).

#### Development of the Attachment Guide

A step-by-step guide detailing app use and accelerometer attachment was developed by the project team. The guide was designed to fit within the flow of the smartphone app, taking the participants through the accelerometer attachment process, from preparing the attachment area to attaching the accelerometer. Once the attachment is complete, participants are guided through the navigation of the app (eg, how to correctly enter work and sleep hours). The guide was assessed in phases 1 and 2.

#### Usability Evaluation of the Smartphone App and Attachment Guide

**Figure 5:**
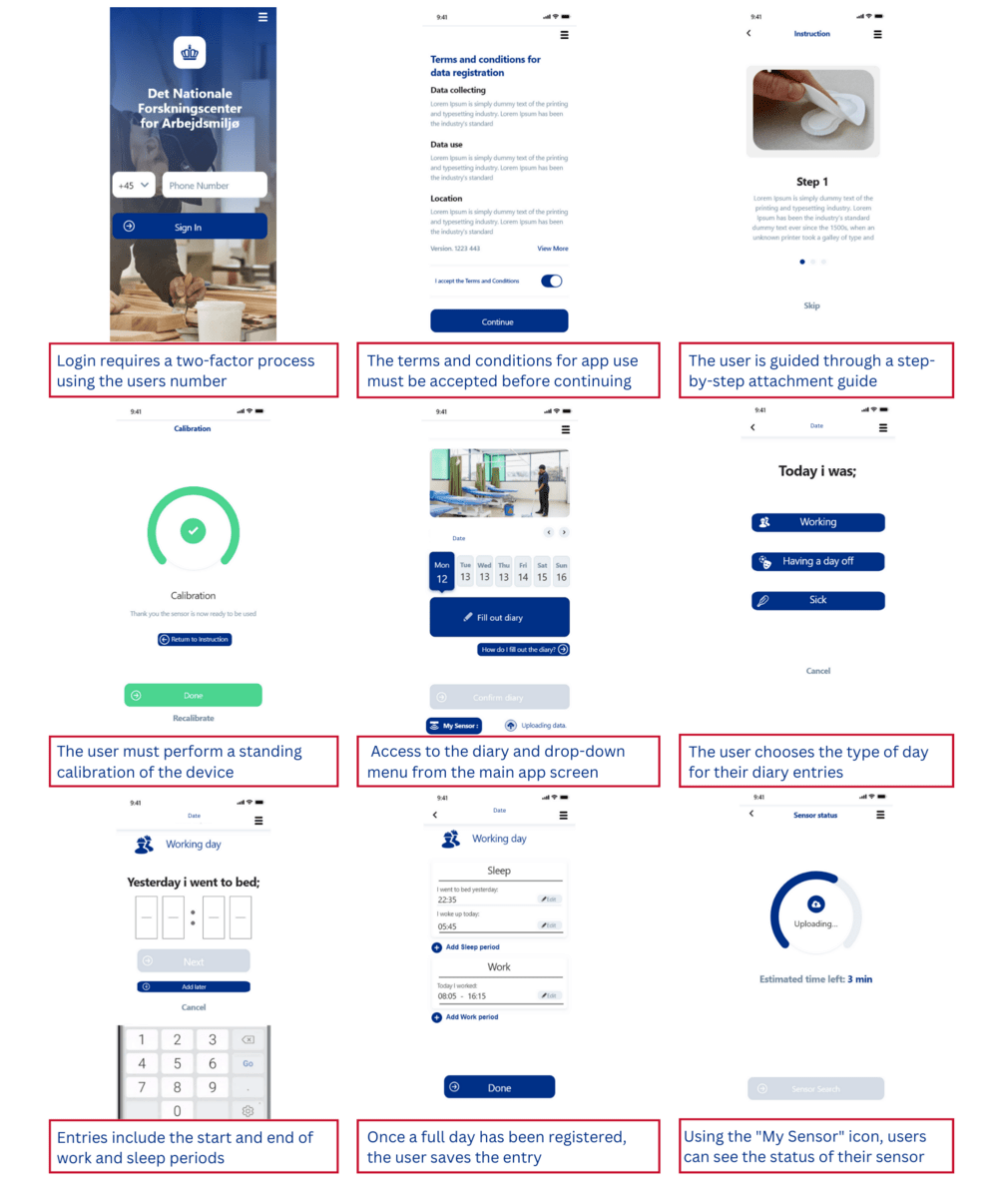
Examples of the app screens used in the phase 2 usability evaluation. From top left corner to bottom right corner; two-factor identification login, terms and conditions for app use, a step-by-step guide for accelerometer attachment, accelerometer calibration, diary entry, and data transfer status verification.

The interactive browser-based app screens, developed in Adobe XD, were presented to 7 participants using the “share screen” function of Microsoft Teams. A think-aloud walkthrough method was used [14], whereby participants complete a task under observation while voicing their thoughts and posing any questions that they might have [15]. Participants were requested to perform tasks related to app login, accelerometer attachment, main screen navigation, work and sleep hour entry, and data transfer status verification (examples shown in Figure 5). An observer took notes of the participants’ comments and categorized the comments using a color-coding scheme. “Red” referred to a critical issue, “yellow” referred to a serious issue, “blue” referred to a minor issue, and “green” if no issue was detected. A critical issue was defined as an issue that inhibits the correct entry of information in the app. A serious issue was defined as an issue that led the participant to make a mistake, from which they were able to recover and continue. A minor issue is defined as a subjective preference not related to the functional performance of the app. This information was passed to the industrial partners and used to create an improved and fully functional smartphone app, for use in the evaluation phase 2.

### Phase 2 Development and Evaluation

#### Integration of the Cloud Infrastructure

Cloud infrastructure from SENS Innovation was integrated into the Motus system, with access restricted to the project team via a code-protected login. Stored data were encrypted according to the industrial standard AES-256. Moreover, data were end-to-end encrypted under transfer to the cloud storage, using a service provided by Amazon Web Services (AWS GDPR Data Processing Addendum). The full integration of the analysis software is a work in progress and data analyses for the generation of participant feedback are conducted offline at the time of writing. The offline feedback was sent to the participant via email.

#### Integration of the Web Application

A web application was adapted, from an existing SENS Innovation web app, to include the logo and color scheme of the National Research Centre for the Working Environment, facilitate participant registration, allow for the assignment and activation of sensors, and support the download of diary and accelerometer data. Access to the web application was again restricted to the project team via a code-protected login.

#### Modification of the Analysis Software

A modified software, built around the source code of the validated Acti4 software [16] and algorithms from the ActiPASS software [17] was developed. This version integrated a validated algorithm for differentiating between sitting and lying down using only a thigh-worn accelerometer [17,18] and a validated algorithm for automatically correcting the reference angle of measurement. The latter is implemented if the reference angle produced by the participant is unlikely (eg, if the participant mistakenly performs the calibration while sitting, instead of standing) [19]. This adjustment accounts for differences in alignment (eg, because of thigh shape or placement position). An algorithm to detect, and adjust for, incorrect orientation of the sensor (if the sensor had been attached “back to front” or “upside down”) was implemented. Finally, an algorithm that removed extended periods of activities or postures during the sleep period which are not classified as lying down was implemented. Modifications were conducted in Matlab.

#### Development of Recruitment Material

Recruitment material was developed by the project group and included an information letter intended for the package that participants would receive [9] and the study web page containing detailed project information, a consent request form, and a registration form. The information letter contained an explanation of what could be expected from the meeting on day 1, during the measurement period, after the measurement period, and during the exit interview. The contact information of the responsible researcher was also provided. The web page provided participants with a detailed account of the project, and the aim of the measurements and allowed them to provide informed consent to participate. They were then directed to a registration form hosted by SurveyXact (Ramboll Group), where they could register their contact phone, email, and postal address.

#### Usability Evaluation of the Motus System

A total of 12 participants completed a 7-day user test of the system. On day 1, in individual web-based meetings, participants were asked to complete 20 tasks related to system use, including registration on the study web page; the procedure for attaching the accelerometer; and using the smartphone app. The think-aloud method was used, and the task completion rate was noted by an observer. Prompting was only provided when a participant was unable to follow the instructions and to indicate which tasks were to be performed after the participant had logged into the smartphone app.

Participants then wore the SENS motion Plus (47 × 22 × 4.5 mm; 7 g) accelerometer on the midsection of their right thigh for 7 days, while registering their work and sleep hours in the smartphone app. Acceleration data were sampled at 25 Hz. After the 7 days, participants completed a semistructured interview aimed at determining the usability of the system and the participants’ opinions on the feedback provided to them (Multimedia Appendix 1). The participants were also asked to provide a rating of usability on the 10-item System Usability Scale [15]. A topic summary thematic analysis was conducted within the essentialist paradigm and analyzed following a semantic approach [20]. Data were transcribed using an intelligent verbatim account of participant responses, which were then assigned to predefined codes (Multimedia Appendix 1) and interpreted with regard to their relation to the subconstructs of usability; learnability, efficiency, memorability, safety, and satisfaction [13]. The overall usability score was calculated by subtracting 1 from the scale position of items 1, 3, 5, 7, and 9 and subtracting the scale position from 5 for items 2, 4, 6, 8, and 10 [15], resulting in a scale ranging from 0 to 100. Then the average usability score across all participants was calculated.

## Results

### Participant Demographics

Participant demographics stratified by group are presented in Table 1. The average age of the groups combined was 45 (SD 11) years and 58% (11/19) were female.

**Table 1:** Demographic characteristics of participants in the two-phase usability evaluation of the Motus system.

		Phase 1 (n=7)		Phase 2 (n=12)
Sex (female), n (%)		4 (57)		7 (57)
Age (years), mean (SD)		49 (10)		43 (11)
Shift work (yes), n (%)		1 (14)		4 (33)
**Occupation, n (%)**				
	Information technology	1 (14)	—	
	Design	1 (14)	—	
	Education and research	1 (14)	—	
	Emergency services, defense, and police	1 (14)	—	
	Construction	1 (14)	1 (8)	
	Administration	2 (29)	1 (8)	
	Childcare and education	—	1 (8)	
	Health and eldercare	—	7 (58)	
	Cleaning	—	1 (8)	
	Engineering	—	1 (8)	
**Education level (years of further education), n (%)**				
	2 years or younger	1 (14)	4 (33)	
	3-4 years	6 (86)	5 (42)	
	5-6 years	—	3 (25)	

### Phase 1 Evaluation Results

The color-coding of notes taken during the think-aloud walkthroughs revealed 13 critical issues, 12 serious issues, and 10 minor issues in the app. Critical issues related exclusively to the entry of diary information, including the registration of multiple shifts or sleep periods, and the registration of sleep periods that started after midnight. A summary of the notes taken can be found in Multimedia Appendix 2.

### Phase 2 Evaluation Results

The task completion rate was 100% for 13 out of 20 tasks (Table 2). Only 36% (4/11) understood the next step after reading the information letter (ie, that they needed to download the app), 17% (2/12) of participants could not locate instructions for diary entries and sensor attachments from the menu on the home screen, and 8% (1/12) could not locate the sensor status icon on the main screen or the contact information on the home screen. Finally, 42% (5/12) of participants could find the sensor status page from the drop-down menu and 50% (6/12) were unable to use the “search for sensor” functionality.

The overall usability rating was 86/100 (SD 9; range 65-95). A summary of the thematic analyses around the subconstructs of usability is presented in Table 3.

**Table 2:** The task completion rate during think-aloud walkthroughs in phase 2, where the usability of Motus was evaluated in free-living settings. Having received a package, including a sensor, patch, and information letter, participants opened the package and followed instructions without prompting. After app login, researchers prompted the performance of further tasks (below).

Task		Completed task (%)	Participant suggestions for improvement
**Website^a^**			There should be an indication when registration is completed
	Find registration link	100	
	Read information and provided consent	100	
**Package^b^**			Clarify how often the app should be used each day
	Read information letter	100	
	Proceed to download the app	36	
**App login**			None
	Type the correct telephone number	100	
	Receive and use a single-use login code	100	
	Accept terms of use	100	
**Sensor attachment**			There should be numbers on the patch indicating the sequence that adhesive covers should be removed
	Read the instructions	100	
	Attach sensor correctly	100	
	Execute correct calibration	100	
**App home screen**			None
	Locate diary entries	100	
	Locate instructions in the drop-down menu	83	
	Locate sensor status	92	
	Locate contact information	92	
**Diary**			Provide clearer instruction on how to register work periods that cross midnight
	Select the correct day	100	
	Fill out work hours correctly^c^	100	
	Fill out sleep hours correctly	100	
**Check sensor status**			Provide a video instruction
	Find the sensor status	42	
	Use the “search for sensor” function	50	
	Find contact information	92	

^a^Seven out of 12 completed registration before the web-based meeting.

^b^One participant opened the package before the web-based meeting.

^c^Two participants held the web-based meeting on a day off, so they did not register work hours on day 1.

**Table 3:** A summary of the analysis of interview data exploring the usability of Motus. The analysis is based on verbatim transcriptions of semistructured exit interviews. Data were assigned to the predefined codes of learnability, efficiency, memorability, safety, and satisfaction (each a subconstruct of usability [13]).

Sub construct	Analysis	Evaluation
Learnability	All participants found the app easy to navigate All participants found the instructions easy to follow	Easy to learn, but attachment instructions could be improved.
Efficiency	3 out of 12 wondered how long the work and sleep time process would take 11 out of 12 used less than 3 minutes on sleep and work time 8 out of 12 had time issues with uploading data	Clarification of the expected daily time burden would be beneficial and the speed of data upload time needs to be improved
Memorability	All participants registered work and sleep time for 7 days 8 out of 12 typically registered hours each morning	Functionality was easy to remember over several days
Safety	4 out of 12 participants found the entry of work and sleep hours confusing	Few errors, mostly related to the entry of atypical work and sleep hours. Issues with the adhesive patch were also noted toward the end of the measurement week (eg, itching, becoming loose)
Satisfaction	10 out of 12 would take part in measurements again 6 out of 12 felt engaged throughout the measurements (n=6)	The app was subjectively pleasing, although a few participants desired more frequent and detailed feedback on their level of activity. Most did not notice wearing the sensor.

Most participants (10/12, 83%) found the information letter and the design of the app easy to use.

It was easy to understand, not complicated and does not need much guidance.

Participant comment regarding instructions

Yes it has helped me, and is not hard to figure out [when red turns to green] for when I had registered correctly.

Participant comment regarding app design

Although a few participants (3/12, 25%) felt they needed more information about how to attach the sensor and a few said they would like animation or video instruction, learnability and efficiency for the majority were high.

Self-reported app use was less than 3 minutes per day. However, half of the participants (6/12, 50%) had issues with the duration required to upload accelerometer data, and a few (3/12, 25%) doubted the app’s reliability as a result.

It went very slow, before it reached 100%. So last night I left it turned on with the sensor on top of [the phone] to be sure it reached a 100% [of uploading of data].

Participant comment regarding data transfer

The upload and the percentage-counter made me unsure if the data had been uploaded correctly.

Participant comment regarding the app functionality

Aside from the duration of data upload, almost half of the participants (5/12, 41%) frequently reported difficulty when registering work and sleep hours for those with atypical work shifts.

I was in doubt about when I slept, since it asks when you slept yesterday. That is the question, which is fine in itself. When I had night shifts, then I would just write approximately, when I got to bed. The thing is, I woke up sometimes during the night and it was not possible to register.

Participant comment regarding difficulty with diary entry

Another participant voiced these concerns as well but came up with a solution to these types of errors.

There could maybe have been a video for how to register when you work all three shifts as a nurse. Possibly show some kind of example of how to register it correctly.

Participant suggestion of a solution to diary entry difficulty

Nevertheless, most participants (11/12, 91%) were confident in both the app and the task of registering work and sleep times.

It was quite easy, since I could go back to [the sleep/work time screen]. Therefore, if I was unsure of anything then I could go back and edit it. It is a great function to have.

Participant comment regarding registration in the app

Most participants (8/12, 66%) stated that they did not feel or notice the sensor during the measurement.

The best experience was having this sensor because I could almost forget that I was wearing something. During the last seven days, I went to the beach, I was swimming in the water, and I did not feel it. I was taking showers, changing clothes, doing exercise, sitting, running, and standing. It was not at all bothering me in any way.

Participant comment regarding wearing the sensor

A couple of participants (2/12, 16%) had issues when working out while wearing the sensor and experiencing itchy or uncomfortable sensations during the last days of measurement.

For half of the participants (6/12, 50%), the patch became loose around the edges and a few reported developing their own solutions (eg, Band-Aids or other adhesives to ensure the sensor stayed on).

I went to the pharmacy to buy a square water-resistant Band-Aid, because it had loosened at one of the roundings at the top of the patch when I took the first shower with it on. I went […] and put that on top of everything else. It was like that for all the 7 days.

Participant comment regarding patch coming loose

Overall, the participants found the system satisfactory to use. A few participants (3/12, 25%) would have liked more features to be made available in the app (eg, the ability to track physical activity daily) and compared the app to other health-tracking apps.

The only thing I thought was a bit strange was that I did not have to register my exercise as with the app I have on my iPhone.

Participant comment regarding comparing to other apps

It is a task I have committed to, and I am not getting any immediate feedback for something that is tangible to me, understanding of my patterns, or using it in some way.

Participant comment regarding missing feedback feature

## Discussion

### Evaluation

We developed a device-based measurement system for physical behavior that removes the need for in-person meetings and the collection of data on work and sleep hours written on paper. The remote participant registration and device attachment were successful, removing the need for participants to meet in person. However, the evaluation highlighted a need for improvement of the information letter, provided in the package sent to participants. Even though participants found the information easy to understand, they clearly needed more direct instruction that they should download the app as the next step, thus facilitating the transition from the information letter to the in-app instruction guide. Participants also indicated the need for early clarification on how much time they were expected to use the app each day.

Removing the need for analog diary data collection was also a success and the smartphone app was widely considered easy to use, easy to learn and remember, and mostly quite efficient when entering diary information (estimated at less than 3 minutes per person). However, a few participants had issues understanding the instructions on how to fill in sleep periods in the app, particularly those who worked atypical shifts.

In addition to a diary entry, the app is also intended to facilitate sensor attachment, calibration, the transfer of accelerometer data, and self-monitoring of sensor status. Although sensor attachment was broadly successful, some participants suggested a further simplification of the attachment guide. A considerable number of participants also experienced slow data transfer, which led to some frustration. Finally, the self-monitoring of sensor status mostly failed, because participants were not able to locate this function.

Other system issues concerned the adhesive patch and the generated feedback. Several participants reported that the adhesive patch became loose toward the end of the measurement, and a number of participants did not consider the feedback generated to be of sufficient detail. A few participants also desired more frequent feedback. However, because this may influence the activity levels of the participant, this suggestion should be considered in relation to the aim of the measurements.

Finally, although we succeeded in streamlining some of the analysis processes, full automation is still a work in progress. Although participants experienced a high degree of usability after interacting with the system, areas in need of improvement were also identified (shown in Textbox 1).

**Suggestions for improvement in these areas.**

**The information letter**

- Provide clearer instructions on when and how to download the app
- Indicate how often the participant should use the app
- Provide an estimate for how long they are expected to use the app each day

**The instructions for diary entry**

- Reformulate current instructions
- Add instructions specific to shift workers

**The sensor attachment guide**

- Simplify the guide
- Explore a video format

**The data transfer speed**

- Instruct participants to regularly keep the app open
- Update the app so that it will not close during upload
- Investigate solutions to improve data transfer speed

**The app**

- Remove the “check sensor status” page and function

**The feedback**

- Provide more detailed feedback
- Provide reference statistics for comparison

### Strengths and Limitations

This usability evaluation was strengthened by the inclusion of user input in an iterative development and evaluation cycle. Moreover, the participant input was gathered in a number of modalities (think-aloud, questionnaire, and interview) and with observation in both controlled and free-living settings. The participants came from a wide range of job groups, typical and atypical shifts, covered a range of age profiles, and were almost equally distributed between males and females, which we believe allowed us to test the usability in a sufficiently wide range of use cases.

There were also a number of limitations to our evaluation. The 2020-2021 COVID-19 pandemic-imposed lockdowns forced think-aloud walkthroughs and interviews to be in a web-based format, which may have influenced responses and may have meant that the observer missed some information [21]. Hopefully, this limitation is outweighed by the effect of removing the participant from the research, as would be the case if Motus were to be implemented in a nontest environment. As the smartphone app was only developed for Android at the time of testing, iOS users were provided with an Android phone for testing (9 out of 12 participants). This may have influenced their experience of using the app as they also had to navigate a new operating system and likely required extra effort to keep track of 2 phones (their personal phone and the one we provided).

### Perspectives

A system with high usability from an end user perspective is a valuable step in moving toward a less burdensome system for device-based measurement of physical behavior that could be applied at scale. Such systems could be continually improved and adapted as the technology develops and new use cases are included (participants with different living conditions or situations, or research with different aims). Early in this development process, 2 aspects will be particularly important. First, there needs to be a preliminary evaluation of the feasibility (ie, does the system work as we expect it to work in reality) to provide an indication of whether the system has real potential for implementation at scale. As is often seen when moving from a small-scale controlled setting to a free-living setting, a system that seems to function on a usability “level” might be met with challenges at the feasibility “level.” A rigorous, perhaps multiple, evaluation or evaluations are desirable. Second, the current evaluation focused on the participant. In the future, it is necessary to invest more resources to increase the system’s usability and ensure feasibility among the personnel administering the measurements. The first step is to improve the automation of the analysis process and develop and evaluate a standard procedure for data collection.

### Conclusions

The usability of the Motus system is good. Motus is user-friendly and is likely to place an acceptable burden on the participants of device-based measurement of physical activity. However, to improve the usability further, a number of issues should be addressed in the further development of the system, such as improving the instruction and information provided and increasing the speed of data transfer. With future improved iterations of the Motus system, device-based measurements could potentially be implemented in large-scale surveillance, providing much-needed data to broaden the evidence base and ensure a solid foundation for policy and practice.

## Appendix

Multimedia Appendix 1 Interview questions.

Multimedia Appendix 2 Summarized notes.

## Data Availability

No public repository has been made, but data is available by contacting the corresponding author.
