# Supplementary material for: A Novel System for the Device-Based Measurement of Physical Activity, Sedentary Behavior, and Sleep (Motus): Usability Evaluation
Source: JMIR Form Res. 2023 Nov 17;7:e48209. doi: 10.2196/48209 (PMC10692873; doi:10.2196/48209)
Supplement: Multimedia Appendix 1 [file formative_v7i1e48209_app1.docx]

**Interview questions S1.** The predefined codes and interview items used in the semistructured exit interview conducted after a 7-day user test of the MOTUS system. Leading questions are indicated by the numbers (eg, 1, 2 3, 4). Probing questions are indicated by the lowercase digits (1a, 1b, 2a, 2b, 2c, 3a, 3b, etc).

| Theme | Item |
| --- | --- |
| Equipment | 1. What did you think about the information you received in the package?   1a. *If the participant was not satisfied with the information: What could have improved the information provided?*   1. How was your experience of wearing the sensor during the measurement period? 2. What could have improved your experience? |
| Diary entries | 1. How often did you fill out the diary? 2. At time in the day, did you normally fill out the diary? 3. How many days did you provide entries for? 4. Do you feel confident that you fill out the information correctly?   4a. *If the participant did not feel confident: What could have made you more confident?*   1. What motivated you to open the app? 2. What do you think could be done to provide further motivation? 3. How would you feel if the app sent you reminders during the week? 4. How often would be happy to receive reminders? 5. At what time of the day would you like to receive reminders? |
| Impression of the app | 1. What did you like best about the app? 2. What did you like least about the app? 3. Was there anything in the app you would change?   3a. *If the participant would make a change: How would you imagine this change?* |
| Burden of participation | 1. On average, how much time did you use per day on participating in the study? 2. On average, how much time did you use reading the instructions? 3. On average, how much time did you use filling out the diary? 4. How often did you notice the sensor? 5. How did you feel about the time you spent on these measurements, if you exclude the time, it took you to participate in our interviews? 6. Would you be willing to participate in such measurements again? If interviews were not involved. |
| Participant support | 1. Did the app help you understand what you had to do during the measurements? 2. Was there anything you were unsure about during your participation?   2a. *If the participant was unsure: How did you find out what you should do?*  2b. *If the participant was unsure: What could have clarified things for you?* |
| Webpage | 1. Did you use the webpage, apart from for registration? 2. Did you find what you searched for on the webpage? |
