# Supplementary material for: A Novel System for the Device-Based Measurement of Physical Activity, Sedentary Behavior, and Sleep (Motus): Usability Evaluation
Source: JMIR Form Res. 2023 Nov 17;7:e48209. doi: 10.2196/48209 (PMC10692873; doi:10.2196/48209)
Supplement: Multimedia Appendix 2 [file formative_v7i1e48209_app2.docx]

**Summarized notes S2.** A summary of the notes taken during the first round of individual think-aloud walkthrough meetings, conducted online with 5 members of group 1. A minor issue is defined as a subjective preference not related to the functional performance of the app. A serious issue is defined as an issue that leads the participant to make a mistake, from which, they were able to recover and continue. A critical issue is defined as an issue that inhibits the correct entry of information in the app.

| **Minor issues**   - Terms and conditions: “I accept” icon was not clear initially. - Guide: Aesthetic inconsistency between screens. - Guide: Return to the previous screen was not clear initially. - Diary entry: 15-minute intervals suggested to improve usability. - Diary entry: Work and sleep entries require too many clicks. - Diary entry: Icon for entering diary information was not clear initially. - Diary entry: “Edit entry” icon was not clear. - Language: Danish to English translation was partial. - Navigation: Not clear where to find the sensor status. - Navigation: User searched for feedback.   **Serious issues**   - Guide: Attachment guide was skipped. - Guide: User was unsure when to use alcohol swab. - Diary entry: Repeated attempts to select the day instead of “Enter entry” icon. - Diary entry: Incorrect date selected initially. - Diary entry: “Enter entry” icon was not found initially. - Diary entry: User accidentally taps outside of the time registration entry is deleted. - Guide: Cannot find instructions for diary entries initially. - Diary entry: Initial entry is incorrect. - Diary entry: Omits end of work time in initial entry. - Diary entry: Instructions for diary entry are unclear. - Diary entry: Initial sleep period entered in reverse. - Diary entry: Not clear how to finalize diary entry, initially.   **Critical issues**   - Guide: App froze on main-screen. - Diary entry: “Enter entry” icon not found. - Diary entry: Entry was deleted unnoticed by user. - Diary entry: Edited entry was not saved automatically. - Diary entry: Could not navigate 24-hour digital format. - Diary entry: Sleep period entered for the wrong date. - Diary entry: Work period entered on wrong date. - Diary entry: User cannot enter a half sick-day - Diary entry: Enters period assuming hours and not a start and end time point. - Diary entry: User cannot register more than one sleep period. - Diary entry: User is unable to register multiple shifts. - Diary entry: User cannot register sleep time that started after midnight - Diary entry: User cannot understand how to register two work shifts. |
| --- |
